# The Influence of the Cancer Microenvironment on the Process of Metastasis

**DOI:** 10.1155/2012/756257

**Published:** 2012-04-18

**Authors:** Andra R. Frost, Douglas R. Hurst, Lalita A. Shevde, Rajeev S. Samant

**Affiliations:** ^1^Department of Pathology, University of Alabama at Birmingham, 1530 3rd Avenue South, Birmingham, AL 35294-1170, USA; ^2^Department of Oncologic Sciences, Mitchell Cancer Institute, University of South Alabama, 1660 Springhill Avenue, Mobile, AL 36604, USA

Metastasis of breast cancer is a multistep process that requires cancer cells to invade stroma at the primary site, gain access to vasculature, survive in the circulation, extravasate into the parenchyma of the secondary site, and survive and proliferate at the secondary site. During each of these steps, the microenvironment surrounding the cancer cells is believed to be an active participant. The cancer microenvironment also varies during the metastatic process. At the primary tumor in the breast, invasive cancer cells are surrounded by fibroblasts, extracellular matrix (ECM), cellular constituents of the vasculature, inflammatory/immune cells, and adipose tissue. Metastasizing cancer cells are exposed to a completely different microenvironment within the circulatory system. The cancer microenvironment at the secondary site is, again, very different from the microenvironment of the breast and varies depending on the sites of metastasis. At the primary and metastatic sites, the interaction between tumor cells and their surrounding milieu is reciprocal; the tumor cells influence the stroma and vice versa, ultimately fueling tumor progression. The papers in this issue discuss the dynamics of the interactions of tumor cells and their microenvironment, detailing how tumor cells manipulate their milieu and, conversely, how the reactive tumor microenvironment influences tumor cell plasticity, invasion, metastasis, and cancer therapy.

Z. I. Khamis et al. provide a thorough summary of the roles of the tumor stroma and tumor microenvironment in the various steps involved in the metastatic process, as well as in the development of breast cancer in their paper *“Active roles of tumor stroma in breast cancer metastasis.”* The authors discuss research findings related to the contribution of various constituents of the tumor microenvironment, including inflammatory cells, fibroblasts, extracellular matrix, and blood vessels, in the metastatic process. They also include a discussion of the signaling pathways utilized by cancer cells to modify the stroma and ECM. This review serves as an excellent overview for this issue.

Two papers in this issue discuss the cancer cells themselves and how characteristics or functions of the cancer cells influence the tumor microenvironment. Just as the microenvironment signals to the cancer cells, the cancer cells alter the microenvironment to promote tumor progression and metastasis. J. E. Chu and A. L. Allan in their paper *“The role of cancer stem cells in the organ tropism of breast cancer metastasis: a mechanistic balance between the “seed” and the “soil?””* have exhaustively summarized the role of the cancer stem cells in determining the organ tropism exhibited by breast cancer cells. Given the fact that metastasis is an inefficient process, the authors make a compelling case for cancer stem cells to be the rare population that is equipped with the necessary armamentarium of traits to successfully metastasize. The paper summarizes the hierarchical role of cancer stem cells within the various subtypes of breast cancer and the phenotypic and functional signatures of breast cancer stem cells. It also puts into perspective the origin of cancer stem cells and their role in conditioning the premetastatic niche. The authors also provide a detailed analysis of the microenvironment of the various metastatic niches encountered by metastatic breast cancer cells, specifically the bone, brain, lungs, liver, and lymph nodes. The paper concludes with a stimulating discussion on the contribution of cancer stem cells to therapeutic resistance taking into account the interactions of the cancer stem cells with the microenvironment.

The review by J. Alsarraj and K. W. Hunter, “*Bromodomain-containing protein 4: a dynamic regulator of breast cancer metastasis through modulation of the extracellular matrix*”, is focused on the activity of bromodomain-containing protein 4 (BRD4) in breast cancer cells. BRD4 functions as an inherited susceptibility gene for breast cancer progression and metastasis and regulates the transcription of select genes through epigenetic mechanisms. Multiple ECM genes are regulated by BRD4 that may lead to changes in the overall structure of the surrounding environment or alter the cell-matrix interactions to promote breast cancer invasion and metastasis. 

The roles of estrogen receptor (ER) signaling and signaling through Toll-like receptors (TLR) in the crosstalk and interactions of breast cancer cells with the tumor microenvironment are the topics of another two papers in this issue. Hormones play a critical role in directing breast cancer progression. Specifically, ER signaling is one of the critical and complex determinants of breast cancer metastasis. S. S. Roy and R. K. Vadlamudi have provided an integrated picture of this specific signaling in the paper “*Role of estrogen receptor signaling in breast cancer metastasis*”. They emphasize the importance of ER-coregulatory proteins and their misexpression in promoting metastasis of ER-positive breast cancer cells. They have discussed possible therapeutic targets to block ER-driven metastasis. Most significantly, this paper brings to notice the importance of defining alternative signaling pathways. Specifically, multiple signaling pathways in addition to estrogen signaling are involved in activating ERs. Hence, combination therapies using both endocrine and nonendocrine agents that block these different pathways may have better therapeutic effects and may delay metastasis. 

D. Bhattacharya and N. Yusuf discuss the data regarding TLR expression in breast cancer and its role in inflammation and cell survival in the tumor microenvironment in *“Expression of Toll-like receptors on breast tumors: taking a Toll on tumor microenvironment.”* The immune system is intricately involved in the process of tumor progression and metastasis and can play key roles in both tumor promotion and tumor suppression. TLRs are critical for innate and adaptive immunity and are expressed on inflammatory cells surrounding the tumor. Recent studies have identified many TLRs expressed by tumor cells that may promote growth and immune evasion. This has led to the emergence of TLR signaling as a potential target for the treatment of various tumors. 

One of the most common sites for the metastasis of breast cancer is to bone. In accordance with this, four papers focus on breast cancer metastasis to bone. B. Y. Reddy et al. put into perspective the role of the microenvironment of the bone in breast cancer metastasis in *“The microenvironmental effect in the progression, metastasis, and dormancy of breast cancer: a model system within bone marrow.”* The heterogeneous composition of the bone microenvironment not only facilitates the growth of breast cancer cells but also supports and protects the tumor cells. There is a bidirectional crosstalk between the cells comprising the bone microenvironment and the metastatic breast cancer cells. While modulation of macrophage function can cause immune suppression, the release of inflammatory cytokines by adipocytes can stimulate tumor cell invasion, and the expression of SDF-1 by the myofibroblasts accelerates tumor cell growth. The contribution of mechanical stress in impacting tumor cell survival, elicitation of angiogenesis, and influencing drug delivery is elegantly summarized. This paper also discusses the role of microenvironment-derived cytokines, chemokines, and miRNA in inducing epithelial-mesenchymal changes and influencing cancer cell quiescence.

D. M. Sosnoski et al. present their findings on the influence of metastases on the levels of a variety of cytokines and growth factors in the bone in their research article *“Changes in cytokines of the bone microenvironment during breast cancer metastasis.”* Using a xenograft model of breast cancer metastases to bone, they demonstrate that the presence of the breast cancer cells in bone changes the normal levels of specific cytokines. Cytokines are important for bone remodeling, hematopoietic processes, and homeostatic balance in the bone. Therefore, by altering cytokine levels in the bone, metastatic breast cancer manipulates the bone microenvironment. 

A complementary perspective on the dynamic dialogue between the stroma and the tumor cells, which impacts metastasis of tumor cells to bone, is provided by E. Bevilacqua et al. in *“RKIP suppresses breast cancer metastasis to the bone by regulating stroma-associated genes.”* This focuses on the metastasis suppressor, Raf Kinase Inhibitory Protein (RKIP), and its ability to influence the tumor microenvironment in the bone. RKIP inhibits breast cancer invasion, intravasation, and bone metastasis via the induction of miRNA let-7, resulting in suppression of the chromatin-remodeling factor HMGA2 and modulation of epithelial to mesenchymal plasticity. The use of a savvy, interdisciplinary approach involving expression arrays from breast cancer patients yielded a deeper understanding of key regulators of genes that form the bone metastasis signature of cancer cells, putting the spotlight on RKIP as a critical regulator of the tumor milieu and impacting the ability of tumor cells to establish bone metastases. 

The bone microenvironment is a fertile soil for metastasis with multiple regulatory molecules affecting growth. Accumulating evidence supports the notion that hedgehog signaling plays a role in breast cancer metastasis to bone. In *“The hedgehog pathway conditions the bone microenvironment for osteolytic metastasis of breast cancer,”* S. Das et al. discuss our current understanding of how the hedgehog signaling pathway alters the bone microenvironment to promote metastatic breast cancer growth. Hedgehog inhibitors may be a viable option for the treatment and/or prevention of breast cancer metastasis to bone. 

Finally, J. W. Rostas and D. L. Dyess have provided the surgeon's perspective of current surgical management of breast cancer in *“Current operative management of breast cancer: an age of smaller resections and bigger cures.”* Consideration of the tumor microenvironment is an emerging frontier in the treatment of breast cancer. The surgeon's primary focus with breast-conserving surgery is obtaining tumor-free surgical margins, but there is a question as to whether residual stromal changes in the breast may affect local recurrence. The authors emphasize that surgical intervention is currently the best hope for definitive cure of breast cancer; however, advances in the treatment of breast cancer as a systemic disease are needed to facilitate long-term cures. Patient-specific molecular diagnosis and the development of targeted chemotherapeutic agents are future hopes for improved survival and will offer the surgeon an opportunity to be more focused and allow easier management of the disease.

*Andra R. Frost* *Andra R. Frost* *Douglas R. Hurst* *Douglas R. Hurst* *Lalita A. Shevde* *Lalita A. Shevde* *Rajeev S. Samant* *Rajeev S. Samant*

